# ABL1-mediated tyrosine phosphorylation of SYCP2 contributes to transcription-coupled homologous recombination and platinum resistance in ovarian cancer

**DOI:** 10.1093/narcan/zcaf031

**Published:** 2025-09-03

**Authors:** Boya Gao, Xudong Wang, Melissa Long, Fengqi Zhang, Yumin Wang, Raj Kumar, Irva Veillard, Bo R Rueda, Oladapo Yeku, Li Lan

**Affiliations:** Department of Molecular Genetics and Microbiology, Duke University School of Medicine, 213 Research Drive, Durham, NC 27710, United States; Massachusetts General Hospital Cancer Center, Harvard Medical School, 13th Street, Charlestown, MA 02129, United States; Massachusetts General Hospital Cancer Center, Harvard Medical School, 13th Street, Charlestown, MA 02129, United States; Department of Molecular Genetics and Microbiology, Duke University School of Medicine, 213 Research Drive, Durham, NC 27710, United States; Massachusetts General Hospital Cancer Center, Harvard Medical School, 13th Street, Charlestown, MA 02129, United States; Department of Molecular Genetics and Microbiology, Duke University School of Medicine, 213 Research Drive, Durham, NC 27710, United States; Massachusetts General Hospital Cancer Center, Harvard Medical School, 13th Street, Charlestown, MA 02129, United States; Massachusetts General Hospital Cancer Center, Harvard Medical School, 13th Street, Charlestown, MA 02129, United States; Division of Hematology-Oncology, Massachusetts General Hospital, 55 Fruit Street, Boston, MA 02114, United States; Department of Medicine, Massachusetts General Hospital, 55 Fruit Street, Boston, MA 02114, United States; Division of Hematology-Oncology, Massachusetts General Hospital, 55 Fruit Street, Boston, MA 02114, United States; Department of Medicine, Massachusetts General Hospital, 55 Fruit Street, Boston, MA 02114, United States; Division of Gynecologic Oncology, Department of Obstetrics and Gynecology, Massachusetts General Hospital, 55 Fruit Street, Boston, MA 02114, United States; Obstetrics, Gynecology and Reproductive Biology, Harvard Medical School, 25 Shattuck Street, Boston, MA 02115, United States; Vincent Center for Reproductive Biology, Department of Obstetrics and Gynecology, Massachusetts General Hospital, 55 Fruit Street, Boston, MA 02114, United States; Division of Hematology-Oncology, Massachusetts General Hospital, 55 Fruit Street, Boston, MA 02114, United States; Department of Medicine, Massachusetts General Hospital, 55 Fruit Street, Boston, MA 02114, United States; Department of Molecular Genetics and Microbiology, Duke University School of Medicine, 213 Research Drive, Durham, NC 27710, United States; Massachusetts General Hospital Cancer Center, Harvard Medical School, 13th Street, Charlestown, MA 02129, United States

## Abstract

Treatment of patients with platinum-resistant ovarian cancer is a major clinical challenge. We found that high expression of a meiotic protein, Synaptonemal Complex Protein 2 (SYCP2), is associated with platinum resistance and tyrosine kinase ABL1 inhibitor sensitivity in ovarian cancer. We demonstrate that tyrosine kinase ABL1 inhibitors inhibit cancer cell proliferation more efficiently in ovarian cancer cell lines with SYCP2 overexpression. Moreover, ABL1 inhibition effectively prevents tumor growth *in vivo*. Mechanistically, we identified a phosphorylation motif [RK]-x(2,3)-[DE]-x(2,3)-Y in SYCP2 and found that abolishing ABL1-mediated phosphorylation of SYCP2 at its tyrosine (Y) 739 within this motif renders ABL1 sensitivity of cancer cells. Importantly, ABL1 and SYCP2 colocalize at sites of R-loops after damage and promote transcription-coupled homologous recombination. Moreover, ABL1-mediated Y739 phosphorylation of SYCP2 promotes function of SYCP2 at sites of R-loops by facilitating RAD51 localization and repair, contributing to ovarian cancer cell survival. Overall, these findings highlight a novel therapeutic mechanism where ABL1 inhibitors induce cell death in platinum-resistant ovarian cancer by impairing transcription-coupled homologous recombination repair.

## Graphical Abstract

**Graphical Abstract:**
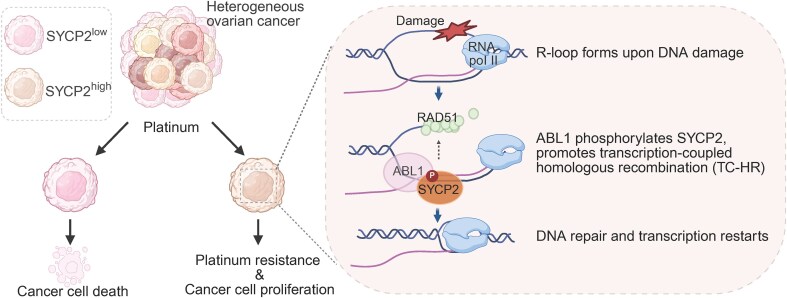

## Introduction

Ovarian cancer is a lethal gynecologic malignancy and ranks as the fourth deadliest cancer among women in the United States [1]. A major contributor to the high mortality is the lack of specific early symptoms, resulting in late-stage diagnoses for 58% of patients, where survival rates are significantly lower [2]. The current standard treatment involves surgical resection, followed by platinum-based chemotherapy; however, despite an initial positive response to treatment, the majority of patients with advanced-stage disease experience recurrence and develop resistance to chemotherapy [3, 4]. Platinum resistance is a particularly pressing issue, as >75% of ovarian cancer patients develop recurrent disease that is resistant to platinum-based therapies [5, 6]. The lack of reliable biomarkers for platinum resistance further complicates the development of effective targeted therapeutics. Identifying novel biomarkers or molecular mechanisms holds promise for improving early detection and addressing platinum resistance in ovarian cancer.

Our recent research has identified Synaptonemal Complex Protein 2 (SYCP2), a meiotic recombination protein, as being upregulated in several cancers, including cervical, breast, and potentially ovarian cancer [7]. SYCP2 facilitates DNA repair through transcription-coupled homologous recombination (TC-HR). In cancer cells, TC-HR helps resolve DNA damage by forming R-loops—RNA–DNA hybrid structures that recruit repair proteins, which enhance cell survival under therapeutic stress. TC-HR may adopt the aberrant activation of meiotic recombination, enhancing cancer cell resilience, promoting therapeutic resistance, and maintaining genome stability in the face of chemotherapy or radiation. Our previous findings further indicate that breast cancer patients with elevated levels of SYCP2 exhibit increased resistance to DNA damage response (DDR) drug treatments, reinforcing SYCP2’s role as a key player in DDR-targeted therapy resistance [7]. While SYCP2’s involvement in R-loop formation has been linked to DDR drug resistance, the precise regulatory mechanisms within the TC-HR pathway remain poorly understood.

Tyrosine kinases are crucial regulators of numerous biological processes, including cell growth, proliferation, differentiation, and apoptosis, largely through the phosphorylation of tyrosine residues on target proteins [8]. Aberrant activation of tyrosine kinases, often due to fusions, mutations, or overexpression, is frequently associated with cancer, making them ideal therapeutic targets. Tyrosine kinase inhibitors (TKIs), such as imatinib, which targets the Bcr-abl (c-abl) ABL1 kinase in chronic myeloid leukemia, have transformed cancer treatment [9]. In ovarian cancer, overexpression of ABL1 has been linked to poor patient outcomes, and ABL1 plays a role in the early stages of the DDR, regulating key processes such as cell cycle progression and DNA repair [10–12]. However, the function of ABL1 within the TC-HR pathway remains largely unexplored, presenting an opportunity to investigate its involvement in ovarian cancer to uncover new therapeutic vulnerabilities.

In this study, we discovered a novel function of ABL1 kinase in ovarian cancer, where it phosphorylates the overexpressed SYCP2. This phosphorylation event plays a critical role in contributing to platinum resistance in ovarian cancer treatment. Specifically, ABL1 colocalizes with SYCP2 at sites of R-loops following DNA damage and phosphorylates SYCP2 at tyrosine residue 739, stabilizing SYCP2 at damaged R-loops. This stabilization enhances TC-HR, facilitating the repair of DNA damage and promoting cancer cell survival under therapeutic stress. Our investigation further suggests that ovarian cancer cells with elevated SYCP2 expression are highly sensitive to ABL1 inhibitors, such as imatinib and dasatinib, which impair this pathway. By targeting ABL1-mediated phosphorylation of SYCP2, we observed disrupted HR, increased R-loop accumulation, and subsequent cell death. Our study underscores the potential of targeting meiotic recombination proteins, like SYCP2, and the unexplored function of ABL1 at R-loops, as novel therapeutic strategies in overcoming drug resistance and improving outcomes for patients with ovarian cancer.

## Materials and methods

### Immunohistochemistry and tissue staining of patient’s tissues

Ovarian tumor specimens were obtained from the Massachusetts General Hospital from patients who provided informed consent to an Institutional Review Board approved banking trial (#07-049). The ovarian cancer samples were collected prior to treatment with chemotherapy and confirmed to be of high-grade serous histology by the institutional pathologists. Of the patients’ tissue samples provided, 12 were platinum sensitive and 16 were platinum resistant or refractory. Resistance/sensitivity was defined by clinical response to platinum-based therapy [progression-free survival (PFS) <6 months = resistant; >12 months = sensitive]. The collected tumor specimens and adjacent normal tissue samples were fixed in 4% paraformaldehyde (PFA) and stored in phosphate-buffered saline (PBS). After sucrose infiltration, samples were filled with Optimal Cutting Temperature mediu (OCT) and ready for cryosection. The immunohistochemistry (IHC) staining, scoring, and analysis followed a triple-blinded manner. The images were captured at 20× and 40× through a digital slide scanner (Aperio CS2, Leica). The counting of positive cells and analysis were performed by one pathologist and one investigator separately in a blinded fashion. Cold sectioning and staining with Ki67 and SYCP2 were performed by the Specialized Histopathology Services at Massachusetts General Hospital. Briefly, samples were fixed, embedded in paraffin, and sectioned into 4 μm thickness. After deparaffinization and rehydration, sections were blocked and incubated with antibodies against SYCP2 (Thermo Fisher Scientific, PA5-67554, 1:20) and Ki-67 (sc-23900, Santa Cruz, 1:200), and then detected using the Dako Envision two-step method of IHC (Carpinteria, CA, USA). The percent positive staining was correlated with either their platinum-sensitive or platinum-resistant/refractory status. The immunohistochemical staining, scoring, and analysis followed a triple-blinded manner. The stratification into High (H), Medium (M), and Low (L) SYCP2 expression is based on the positive staining results, we put the results into 0–4 categories as following: 0: <5%; 1: 6%–25%; 2: 26%–50%; 3: 51%–75%; and 4: >76% staining. SYCP2 low group contains categories 0 and 1, SYCP2 medium group contains category 3, and SYCP2 high group contains category 4.

### Database

Public RNAseq data for cell lines were obtained from the CCLE (Cancer Cell Line Encyclopedia) project (https://portals.broadinstitute.org/ccle) and GDSC project (https://www.cancerrxgene.org/). All expression data were processed to TPM (transcripts per million) by using Python. Expression data from CCLE were normalized by using ln (TPM + 1). RNA seq data of the gene expression are available for download at TCGA (https://www.cancer.gov/), GTeX (https://gtexportal.org/), TARGET (https://ocg.cancer.gov/programs/target/data-matrix), and treehouse (https://treehousegenomics.soe.ucsc.edu/public-data/).

### Cell culture, plasmids, siRNAs, and chemicals

SKOV3 MUC 16+, A2780, COV362, and OVCAR4 were cultured in RPMI (Roswell Park Memorial Institute) 1640 medium (Sigma, R8758) and U2OS-TRE, U2OS-Tet-DR-GFP, HCC1954, HeLa, MDA-MB-231, and OVCAR8 in Dulbecco’s modified Eagle medium (DMEM, Lonza, Catalog#12-604F) with 10% (v/v) fetal bovine serum (FBS, XY Cell Culture, FBS-500) at 37°C, 5% CO2. The U2OS-TRE cells and U2OS-Tet-DR-GFP cells have been described in previous articles [13, 14]. Plasmids pBROAD3/TA-KR, TetR-KR, TA-Cherry, and TetR-Cherry were used in our previous study [13]. pEGFP-SYCP2 and pEGFP-C3 M1 were cloned into pEGFP-C3 vector with KpnI and BamHI. pEGFP-Y739A was created by mutagenesis of pEGFP-C3 M1 using KOD -Plus- Mutagenesis Kit (TOYOBO, SMK-101) acc500) at 37°C, 5%ording to the manufacturer’s instruction. Plasmids were transfected by Lipofectamine 2000 (Invitrogen, 11668019) using a standard protocol. RNase H1 WT and D210N mutant (both HA tagged) were from our previous publication [15]. Small interfering RNAs (siRNAs) were transfected with Lipofectamine RNAiMax (Invitrogen, 13778150) 48–72 h before analysis. The siRNAs used in this study were siSYCP2 (Integrated DNA technologies, 1#, GUCCAAGGAAUCAUGAUGAACUUAA; 2#, TGGCATGCTTGGAGACAAA), and siABLl (Integrated DNA technologies, 1#, GACUUAGAUUGAAGAAACU; 2#, CCUUUGAUGCUUACAAACU).

Dasatinib (SML2589, Sigma), imatinib (CDS022173, Sigma), nilotinib (CDS023093, Sigma), cisplatin (Sigma, 1134357), PARPi olaparib (AZD2281/Ku-0059436, Sellekchem, S1060), irinotecan hydrochloride (CPT11, Sigma, I1406), DNA-PK Inhibitor (6-nitroveratraldehyde, NU7441, 260960, Sigma), ATM inhibitor (Ku-55933, SML1109, Sigma), and ATR inhibitor (NU6027, 189299, Sigma) were used at indicated dose.

### Cell survival assay

Approximately 500 ovarian cancer cells were seeded in each 6-cm dish 24–48 h post siRNA or plasmids transfection. Cells were incubated with RPMI-1640 medium (10% FBS) containing the indicated dose of chemicals as described in the manuscript for 10–14 days. Colonies were then fixed and stained with 0.3% crystal violet in methanol and the number of colonies was counted manually.

### KillerRed activation, immunofluorescence staining, and microscopy

U2OS-TRE cells were cultured in 3.5-cm glass-bottom dishes (P35GC-1.5-14-C, MatTek; Ashland, MA, USA). Cells were exposed to a 15-W Sylvania (Wilmington, MA, USA) cool white fluorescent bulb in a stage UVP (Uvland, CA) for KillerRed protein activation, and then recovered before live-cell observation or fixation. The exposure and recovery time are described in the manuscript.

For the immunofluorescence staining, the recovered cells were washed by cold PBS (BE17-516F) three times, followed by fixation with 4% PFA (Affymetrix, 19943 1 LT) for 10 min at room temperature. Then, the cells were washed three times by PBS and followed by permeabilization using 0.2% Triton X-100 in PBS for 10 min, and washed three times by PBS. The cells were blocked by 5% bovine serum albumin (Sigma, A-7030) in PBS for 1 h at room temperature. Primary antibodies were diluted in blocking buffer and incubated with cells for overnight at 4°C. Cells then were washed three times with 0.05% phosphate-buffered saline with Tween-20 (PBST) and incubated with secondary antibodies for 1 h at room temperature, including Alexa Fluor 405/488/594 goat anti-mouse/rabbit IgG conjugate (Abcam, 1: 500). Finally, they were washed three times by 0.05% PBST. The primary antibodies used in this manuscript are SYCP2 (PA5-67554, Thermo Fisher Scientific, 1:500), RAD51 (ab63801, Abcam, 1:100), S9.6 (ENH001, Kerafast, 1:200), and c-Abl (sc-56887, Santa Cruz, 1:100).

S9.6 staining was described in our previous article [15]. Briefly, after fixation, the cells were incubated in buffer [10 mM Tris–HCl, 2 mM ethylenediaminetetraacetic acid (EDTA), pH 9] on a heating block at 95°C for 20 min. Then, the dish was cooled, washed three times with PBS, and treated with RNase A (100 μg/ml) in buffer (5 mM EDTA, 300 mM NaCl, 10 mM Tris–HCl, pH 7.5) for 15 min at room temperature. Then, the cells were washed and blocked, followed the standard immunofluorescence protocol as mentioned above.

The images were acquired using the Olympus FV1000 confocal microscopy system (Cat. F10PRDMYR-1, Olympus) and FV1000 software. Foci-positive cells were quantified manually using confocal microscopy and ImageJ analysis. Cells were considered foci-positive if the ratio of mean foci intensity over background exceeded 1.5, based on colocalization with TA-KR-induced damage sites. Quantification was performed from three independent experiments with 30 individual cells analyzed per group.

### Tet-DR-GFP reporter assay

U2OS Tet-DR-GFP cell line and I-*SceI*-T2A mCherry are described previously [14]. Briefly, 1 day prior to transfection, U2OS Tet-DR-GFP cells (0.4 × 10^6^) were seeded in DMEM complete medium (10% FBS) in six-well plate. Five micrograms of I-SceI-T2A mCherry was transfected following the standard protocol described above. Eight hours post transfection, cells were trypsinized and replated in two individual 6-cm plates, with one supplemented with 1 μg/ml doxycycline (Dox) and one with vehicle. After 62 h, cells were harvested by trypsinization. A small fraction of cells was analyzed by fluorescence-activated cell sorting (FACS) to determine transfection efficiency and HR efficiency in Dox-treated samples. Transfection efficiency was normalized using mCherry, which is coexpressed with the I-SceI endonuclease. Only GFP (green fluorescent protein)-positive cells within the mCherry-positive population were quantified. The remaining cells were subjected to genomic DNA isolation using the PureLink Genomic DNA Mini Kit (Invitrogen) according to the manufacturer’s instruction. For qPCR (quantitative PCR)-based HR analysis, genomic DNA was diluted to 50 ng/ml in genomic DNA elution buffer, and 75 ng genomic DNA was used in each 15-μl qPCR reaction. The qPCR was performed with the QuantiNova SYBR Green PCR Kit (Qiagen) on StepOnePlusTM Real-Time PCR System (Applied Biosystems). GFP expression was normalized to the internal control gene 36B4. For all qPCR-based quantifications of HR efficiency, transfection efficiency was assessed in parallel by FACS for mCherry-positive cells (I-SceI-T2A-mCherry expression), and only samples with comparable transfection efficiencies were analyzed. This is consistent with the protocol established by Ouyang *et al.* [14].

Primers used in the assay are same with the previous article [14]. Repaired *sceGFP* (wild-type *eGFP*): GFP_Fwd: GGGCGATGCCACCTACG; GFP_Rev: GGTGTTCTGCTGGTAGTGGTCG. For a reference genomic locus (36B4): 36B4_Fwd: CAGCAAGTGGGAAGGTGTAATCC; 36B4_Rev: CCCATTCTATCATCAACGGGTACAA.

### Western blots and RT-qPCR

For western blot analysis, protein preparation was performed with lysis buffer [25 mM Tris–HCl, pH 7.4, 250 mM NaCl, 1% NP-40, 1% sodium deoxycholate detergent, 0.1% sodium dodecyl sulfate (SDS), protease inhibitor (Roche)]. Then, the samples were boiled at 95°C for 5–8 min in SDS loading buffer, subjected to electrophoresis in 10%–12% SDS–polyacrylamide gels, and transferred to polyvinylidene difluoride membranes. The membrane was blocked with 5% nonfat milk in PBS for 1 h at room temperature. The primary SYCP2 antibody (LS-C386874, LifeSpan BioSciences, 1:1000) and c-Abl (sc-56887, Santa Cruz, 1:100) were diluted in blocking buffer and incubated at 4°C overnight, followed by HRP (horseradish peroxidase)-conjugated secondary antibody (1:10 000) incubation at room temperature for 1 h, and then the membranes were washed in 0.1% PBST three times. Chemiluminescent HRP substrate was purchased from Millipore (Cat#: WBKLS0500; Burlington, MA, USA). Images were acquired in the Bio-Rad (Hercules, CA, USA) Universal Hood II machine with corresponding ImageLab software.

For RT-qPCR, total RNA from cell samples was isolated with Purelink RNA Minikit (Invitrogen). The complementary DNA was synthesized using 500 ng of purified RNA with Quantinova Reverse Transcription Kit (Qiagen). The following primers were used: SYCP2-Forward: TTGGAAAAGGGACAGCCAAG; SYCP2-Reverse: GGTTGCTTTTCGTGGAAGTCTG. GapDH-Forward: TTCACCACCATGGAGAAGGC; GapDH-Reverse: TCTCATGGTTCACACCCATGAC. The qPCR was performed with the QuantiNova SYBR Green PCR Kit (Qiagen) on StepOnePlusTM Real-Time PCR System (Applied Biosystems). Reactions were biologically triplicated independent experiments. The results of the expression levels were normalized based on the housekeeping gene GapDH.

### Colony-forming assay

Approximately 350 cells were replated on 6-cm dishes 24–48 h after siRNA transfection. Cells were incubated in DMEM (10% FBS) for 7 days. Colonies were stained with 0.3% crystal violet/methanol and counted. Each experiment was performed three times, and the standard error mean (SEM) was calculated and indicated in the graphs. Results were normalized for plating efficiencies.

### Mouse xenograft model and FACS analysis

SKVO3-MUC16+ cells (1 × 10^7^ cells) were injected intraperitoneally into 16 female NSG mice between 6 and 12 weeks of age. The mice were then randomly assigned into two groups (eight mice per group). Seven days after tumor inoculation, mice were treated with dasatinib by oral gavage on 5 days on, 2 days off schedule for 4 weeks or with placebo following the same schedule. At 21 days post tumor cells injection, two mice from each group were euthanized and subjected to peritoneal wash and the remaining mice from each group were monitored for disease progression and survival analysis. All mice were monitored for survival and were euthanized when showing signs of distress or significant ascites. All animal experiments were approved by and conducted in accordance with the guidelines established by the Institutional Animal Care and Use Committee at the Massachusetts General Hospital (protocol # 2018N000207). Ambient temperature was controlled at 18–26°C, relative humidity was 40%–70%, and 12 h light/12 h dark light-change cycle.

For FACS analysis, peritoneal cells were pelleted and washed three times with FACS buffer (PBS + 2.5% FBS). Cells were resuspended with fluorochrome-conjugated Annexin V and 7-AAD dye according to the manufacturer’s instructions (BD, Cat # 559763). The cells were subsequently washed three times with cold FACS buffer prior to FACS analysis.

## Results

### High SYCP2-expressed ovarian cancer is especially sensitive to tyrosine kinase ABL1 inhibitors

**Figure 1.**
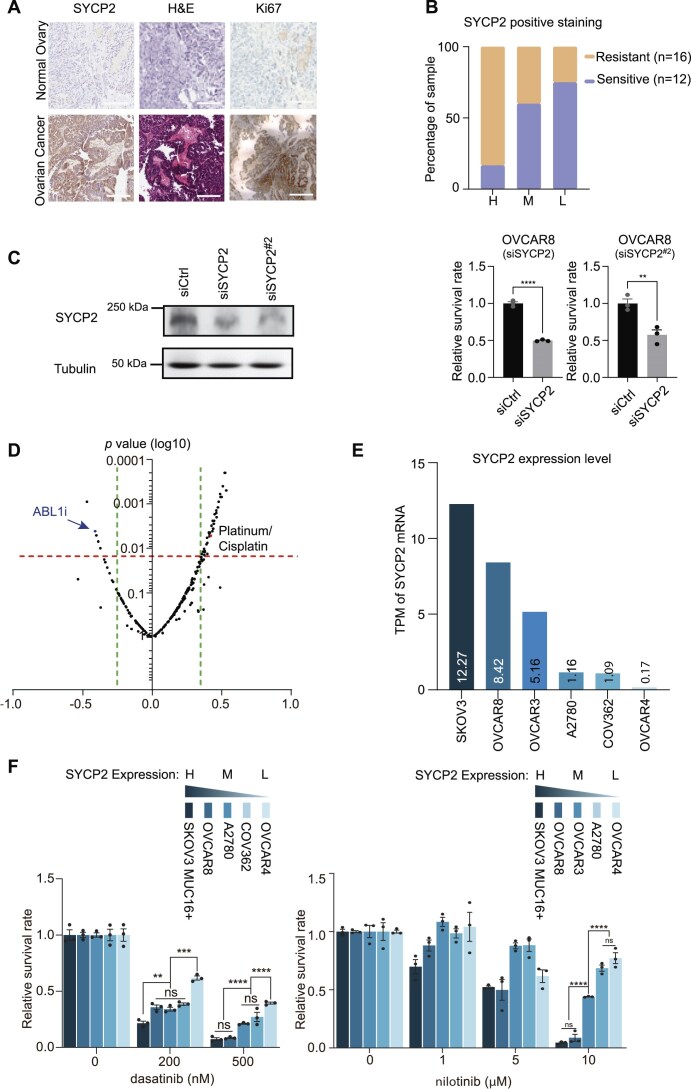
High SYCP2-expressing ovarian cancer is especially sensitive to ABL1 inhibitors. (**A**) IHC staining of SYCP2, H&E, and Ki67 in ovarian cancer and adjacent normal ovary tissues. Representative images from one patient sample are shown (*n* = 28). (**B**) Quantification of SYCP2 IHC staining in 28 ovarian cancer patients (*n* = 28; 16 platinum resistant and 12 platinum sensitive). Patients were categorized based on SYCP2 staining levels into three groups: SYCP2-L (0%–25% positive cells, intensity –/ +), SYCP2-M (26%–50%, intensity ++), and SYCP2-H (>50%, intensity ++/+++) as determined by blinded pathologist scoring. Platinum sensitivity was defined by clinical PFS: >12 months = sensitive, <6 months or progression on therapy = resistant. (**C**) Colony formation assay and immunoblot of OVCAR8 cells transfected with siControl or two distinct SYCP2 siRNAs. Cell viability was quantified in three independent replicates (*n* = 3, mean ± SD), confirming consistent reduction in survival upon SYCP2 knockdown. (**D**) Volcano plot showing Pearson correlation between SYCP2 expression and IC_50_ of 251 drugs in breast cancer cell lines using GDSC and CCLE datasets. ABL1 inhibitors (ABL1i) including imatinib and dasatinib showed significant negative correlation with SYCP2 expression (left side), while platinum-based drugs showed positive correlation (right side). Green dotted lines indicate *P* = .01 and .001 thresholds. (**E**) Expression levels of SYCP2 mRNA (TPM) in six ovarian cancer cell lines (SKOV3, SKOV3-MUC16+, OVCAR8, OVCAR3, A2780, COV362, OVCAR4). Expression data were obtained from CCLE. (**F**) Relative survival rates of ovarian cancer cell lines (categorized as SYCP2-H, -M, or -L) treated with increasing concentrations of ABL1 inhibitors dasatinib (left) or nilotinib (right). SYCP2-H cell lines (SKOV3-MUC16+, SKOV3) were significantly more sensitive to ABL1 inhibitors compared to SYCP2-L lines (COV362, OVCAR4). Data shown are from three biological replicates (*n* = 3, mean ± SD). Statistical significance was assessed using unpaired two-tailed Student’s *t*-test. *P < .05, **P < .01, ***P < .001, ****P < .0001.

SYCP2 is a key component of the synaptonemal complex, a proteinaceous structure essential for chromosome pairing and synapsis during meiosis [16, 17]. It plays a pivotal role in facilitating early meiotic recombination and ensuring accurate homologous chromosome pairing [18]. Previously, we observed that SYCP2 is significantly overexpressed specifically in ovarian and breast cancer patients, where its elevated levels are strongly associated with poorer clinical outcomes and prognosis [7]. To confirm the elevated expression of SYCP2 in ovarian cancer, we performed IHC staining on 28 patient samples. SYCP2 is overexpressed in tumor samples compared to adjacent benign tissues (*P***<**.01, Fig. 1A). Patients were stratified into Low (L), Medium (M), or High (H) SYCP2 expression groups based on IHC scores categorized from 0 to 4: 0: <5%; 1: 6%–25%; 2: 26%–50%; 3: 51%–75%; 4: >76%. L = 0–1, M = 2–3, H = 4. Clinical platinum response was used to further classify patients into platinum sensitive (PFS >12 months) and platinum resistant or refractory (PFS <6 months or progression on treatment). We found that patients in the SYCP2-H group were more frequently platinum resistant, whereas the SYCP2-L group was largely platinum sensitive (Fig. 1B). To evaluate SYCP2 functionally, we performed SYCP2 knockdown in ovarian cancer cell lines with high SYCP2 expression, including OVCAR8 and SKOV3 (both WT and MUC16+). We used two independent siRNAs targeting distinct regions of SYCP2, and in all cases, SYCP2 depletion significantly reduced cell viability compared to control siRNA (Fig. 1C and Supplementary Fig. S1A). To identify potential treatments to counteract SYCP2-induced platinum resistance, we conducted a drug screening using the Genomics of Drug Sensitivity in Cancer (GDSC) database. We found that ABL1 inhibitors, such as imatinib and dasatinib, exhibited a negative correlation with platinum resistance, suggesting they could serve as potential treatment options for ovarian cancer cells with overexpressed SYCP2 (Fig. 1D). In Fig. 1D, the *X*-axis represents the Pearson correlation coefficient between SYCP2 expression and drug sensitivity (the half maximal inhibitory concentration, IC_50_values) for each drug across a panel of ovarian cancer cell lines and patient datasets (TCGA and CCLE). Each dot corresponds to a single drug, with negative values indicating increased sensitivity and positive values indicating increased resistance in relation to SYCP2 expression. Further analysis revealed that the IC_50_ of imatinib (Pearson *r* = –0.5342) is negatively correlated with SYCP2 expression, in contrast to the positive correlation observed with olaparib (Pearson *r* = 0.4736) (Supplementary Fig. S1B). Importantly, recent FDA withdrawals of PARP inhibitors, such as olaparib, for late-line treatment of ovarian cancer due to decreased survival underscore the need for alternative therapeutic options [19]. This context enhances the relevance of our drug screening approach and indicates that targeting the ABL1-SYCP2 axis warrants further investigation (Supplementary Fig. S1B). To evaluate the inhibitory effects of ABL1 inhibitors on ovarian cancer cells with elevated SYCP2 expression, we measured expression of SYCP2 in several ovarian cancer cell lines (Fig. 1E). Our previous studies confirmed that expression of SYCP2 messenger RNA (mRNA) tightly correlated with its protein expression [7]. Here, we treated ovarian cancer cell lines exhibiting varying mRNA levels of SYCP2 with three FDA-approved TKIs targeting ABL1—dasatinib, nilotinib, and imatinib. We observed both dose-dependent and an SYCP2-expression-dependent decrease in survival rates in all three ABL1 inhibitors (Fig. 1F and Supplementary Fig. S1B). Furthermore, combination of dasatinib with different cancer treatments—cisplatin, IR, Olaparib, and CPT11—resulted in significantly reduced cancer cell survival rates compared to single-agent treatments alone (Supplementary Fig. S1C and D), indicating function of ABL1 may be critical to contribute to cell survival by promoting DNA repair.

### ABL1 inhibitors attenuate the SYCP2 foci at sites of DNA damage

**Figure 2.**
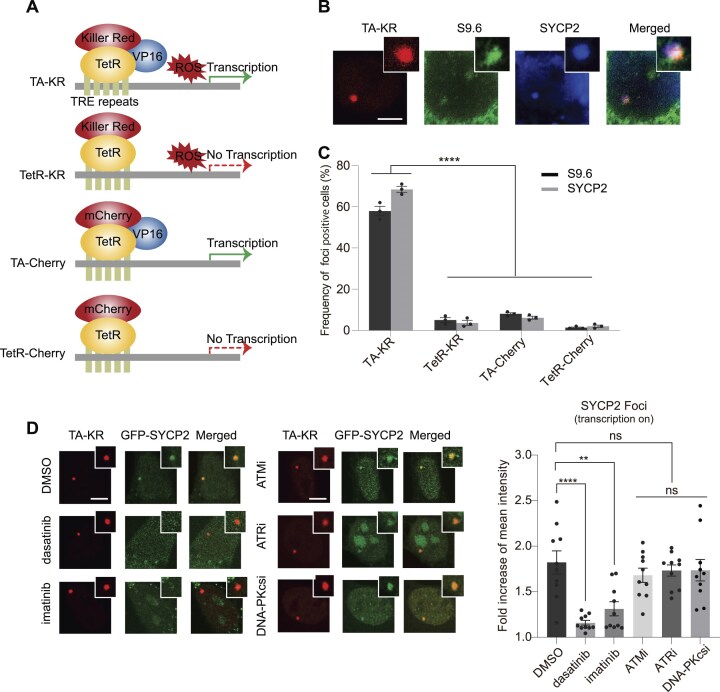
ABL1 inhibitors attenuate SYCP2 recruitment to transcriptionally active loci under ROS-induced DNA damage. (**A**) Schematic of the DART assay. TA-KR (TetR-KillerRed-VP16) drives local transcription and generates ROS at the TRE locus upon light activation. TetR-KR (no VP16) or mCherry fusion constructs serve as negative controls for transcription or damage. (**B**) Representative images of SYCP2 and S9.6 immunofluorescence staining following TA-KR-induced damage in U2OS-TRE cells. Scale bars, 5 μm. (**C**) Quantification of SYCP2 and S9.6 foci frequency in cells expressing TA-KR, TetR-KR, TA-Cherry, or TetR-Cherry (*n* = 3 biological replicates; mean ± SEM). Cells were fixed 30 min post-light activation. SYCP2 colocalizes with S9.6 at TA-KR sites, consistent with its role at damaged R-loops. (**D**) U2OS-TRE cells transfected with TA-KR and GFP-SYCP2 were treated with dimethyl sulfoxide or the indicated kinase inhibitors: 500 nM dasatinib, 1 μM imatinib (ABL1 inhibitors), 10 μM ATM inhibitor (Ku-55933), 10 μM ATR inhibitor (NU6027), and 1 μM DNA-PKcs inhibitor (NU7441). SYCP2 foci formation at TA-KR sites was assessed by confocal microscopy 30 min after light activation. ABL1 inhibitors, but not ATM, ATR, or DNA-PKcs inhibitors, significantly reduced SYCP2 localization to damage sites (*n* = 10 cells per condition; mean ± SEM). Statistical analysis was performed using unpaired two-tailed Student’s *t*-test. *P < .05, **P < .01, ***P < .001, ****P < .0001.

Building upon our previous findings that SYCP2 promotes homologous recombination (HR) by stimulating R-loop formation at sites of DNA damage [7], we sought to understand the observed correlation between SYCP2 expression levels and sensitivity to ABL1 inhibitors in ovarian cancer cells. To investigate SYCP2’s response to DNA damage within actively transcribed regions, we employed the DNA damage at RNA transcription (DART) assay [13, 20]. In the tetracycline-responsive elements (TREs) and a transcription cassette-integrated U2OS cell line at one genomic locus, we expressed a fusion protein comprising TetR, KillerRed, and the transcriptional activator VP16 (TA-KR). This fusion protein TA-KR binds to TREs within the integrated cassette in the U2OS cells to activate local transcription [13, 20]. Upon light activation, KillerRed generates reactive oxygen species (ROS) and induces DNA damage at the TA-KR locus, enabling us to analyze the local DDR (Fig. 2A). R-loops are enriched at sites of TA-KR upon damage induction [15], moreover, costaining for SYCP2 and S9.6 at sites of TA-KR confirmed our previous finding that SYCP2 localizes to damage-induced R-loops at sites of TA-KR at actively transcribed regions (Fig. 2B). In contrast, TetR-KillerRed without VP16, as well as TetR or TA-Cherry without KR-induced damage, served as controls to validate transcription or damage-dependency of localization. The frequency of SYCP2 localization mirrored R-loop accumulation in the DART system, supporting a role for SYCP2 in responding to transcription-coupled DNA repair (Fig. 2C).

Given that many DNA kinases are important to regulate DDR [21], we then sought to determine upstream kinases that might regulate SYCP2 localization to R-loops. We tested a panel of DNA kinase inhibitors, including ATM inhibitor (ATMi), ATR inhibitor (ATRi), DNA-PKcs inhibitor (DNA-PKcsi), and two ABL1 inhibitors—dasatinib and imatinib. Notably, only the ABL1 inhibitors significantly reduced SYCP2 localization to DNA damage sites, suggesting that ABL1 specifically regulates SYCP2 at R-loops (Fig. 2D). Further analysis confirmed that ABL1 inhibitors do not alter the upstream expression of SYCP2 (Supplementary Fig. S2A and B), indicating that ABL1’s influence is specific to SYCP2’s recruitment to DNA damage sites rather than its expression levels. These results highlight ABL1’s role in modulating SYCP2 localization to R-loops, providing insight into the mechanisms underlying DNA repair regulation in ovarian cancer cells.

### ABL1 inhibitors affect SYCP2 recruitment at DNA damage sites through ABL1-mediated Y739 phosphorylation in SYCP2

**Figure 3.**
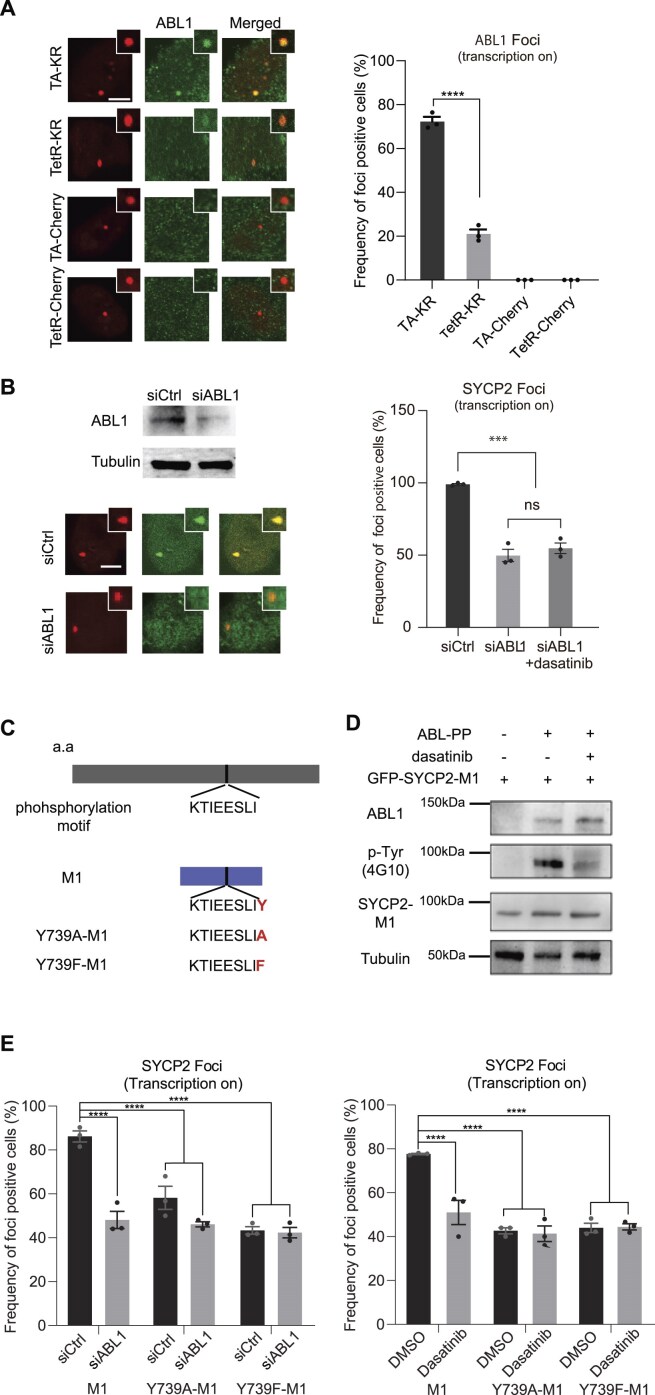
ABL1 affects SYCP2 recruitment to DNA damage sites through ABL1-mediated phosphorylation at Y739. (**A**) U2OS-TRE cells transfected with TA-KR, TetR-KR, TA-Cherry, or TetR-Cherry were stained for ABL1. ABL1 is preferentially recruited to TA-KR damage sites in a transcription-dependent manner. Quantification of ABL1-positive foci (*n* = 3 biological replicates; mean ± SD). (**B**) U2OS-TRE cells were treated with siABL1 and transfected with TA-KR and GFP-SYCP2. SYCP2 foci formation was quantified with or without 200 nM dasatinib. ABL1 knockdown significantly reduces SYCP2 foci at TA-KR sites, and dasatinib does not further reduce this effect (*n* = 3 replicates; mean ± SEM). (**C**) Schematic of SYCP2 phosphorylation motif (KTIEESLY) and Y739A/Y739F mutants in the M1 domain. (**D**) Immunoblot showing tyrosine phosphorylation of SYCP2-M1 by constitutively active ABL1 (ABL-PP), which is abolished by dasatinib treatment. Tyrosine phosphorylation was detected by anti-pTyr (4G10). (**E**) SYCP2 knockdown cells were rescued with wild-type M1 or phospho-deficient mutants (Y739A-M1 or Y739F-M1). ABL1 knockdown or dasatinib significantly reduced SYCP2 localization only in wild-type M1-expressing cells, not in mutant-expressing cells. Data show frequency of SYCP2 foci-positive cells (*n* = 3 groups; mean ± SEM). Statistical analysis was performed using unpaired two-tailed Student’s *t*-test. *P < .05, **P < .01, ***P < .001, ****P < .0001.

ABL1 is a ubiquitously expressed nonreceptor tyrosine kinase that plays a critical role in various cellular processes, including cell proliferation [22], differentiation [23], and apoptosis [23]. One of its notable functions is involved in DDR, where it regulates the phosphorylation of proteins involved in RNA-dependent DDR pathways, e.g. RAD52 [24]. Despite the established involvement of ABL1 in DDR, several key aspects remain unknown, including whether ABL1 localizes to sites of DNA damage and its specific regulatory mechanisms within the pathway. Based on our previous findings that ABL1 is required for SYCP2 localization, We found that ABL1 was preferentially recruited to TA-KR sites following damage, but this recruitment was significantly reduced after RNase H1 treatment, which dissolves RNA–DNA hybrids (Supplementary Fig. S2C), indicating ABL1’s involvement at damaged R-loops (Fig. 3A).This provides evidence that ABL1 is recruited to sites of DNA damage, in the presence of R-loops, offering novel insights into its potential role in the DDR.

We further tested the recruitment of SYCP2 at TA-KR in cells with siABL1 with or without ABL1 inhibitor (dasatinib) treatment. Our results showed that reducing ABL1 levels significantly decreased SYCP2 localization to DNA damage sites (Fig. 3B). Importantly, SYCP2 knockdown did not affect ABL1 foci formation at these sites (Supplementary Fig. S2D), suggesting that ABL1 recruitment is SYCP2-independent. Furthermore, giving dasatinib to ABL1-deficient cells did not further diminish SYCP2 localization, indicating that dasatinib’s effect is primarily mediated through ABL1 inhibition (Fig. 3B), suggesting that ABL1 directly regulates SYCP2 function at R-loops. To elucidate how ABL1 regulates SYCP2 response at damaged R-loops, we analyzed the amino acid sequence of SYCP2 and identified potential phosphorylation motif by ABL1. SYCP2 is a large protein comprising 1530 amino acids. We divided it into several fragments to explore its regulation in R-loops and TC-HR. Our previous study identified the middle (M) domain of SYCP2 as crucial for its localization to R-loops, specifically highlighting the M1 fragment (amino acids 492–1035) as necessary for this function [7]. Within this M1 domain, we discovered a potential phosphorylation motif at tyrosine 739 (Y739), which may be targeted by ABL1 (Fig. 3C). To directly test whether ABL1 phosphorylates SYCP2, we performed phospho-tyrosine blotting of SYCP2-M1 in the presence of constitutively active ABL1 (ABL1-PP). We observed robust tyrosine phosphorylation of GFP-tagged SYCP2-M1, which was abrogated by dasatinib treatment (Fig. 3D). This effect occurred without changes in protein expression, confirming that ABL1 kinase activity directly mediates SYCP2 phosphorylation at tyrosine residues (Fig. 3D). To assess the role of tyrosine 739 (Y739) in SYCP2’s function at damaged R-loops, we generated two phosphorylation-defective mutants: Y739A-M1 and Y739F-M1. Both mutants exhibited a significant reduction in localization to damaged R-loops, indicating the importance of Y739 phosphorylation for SYCP2’s recruitment to these sites. Furthermore, in cells expressing these mutants, neither ABL1 knockdown nor dasatinib treatment led to additional decreases in SYCP2 localization, suggesting that ABL1-mediated phosphorylation at Y739 is crucial for SYCP2’s function at R-loops (Fig. 3E). Notably, we observed that ∼50% of SYCP2 localization to DNA damage sites is dependent on ABL1 phosphorylation, indicating that ABL1 promotes but is not solely responsible for SYCP2 recruitment. This partial dependence suggests an additional, ABL1-independent mechanism of SYCP2 localization. Consistent with our previous work [7], SYCP2 exhibits intrinsic affinity for RNA–DNA hybrids, which may account for this residual localization.

These findings confirm that ABL1 regulates SYCP2’s function at damaged R-loops through phosphorylation at tyrosine 739 (Y739). Inhibition of ABL1, either by knockdown or treatment with dasatinib, disrupts this phosphorylation, thereby impairing SYCP2’s role in the DDR.

### ABL1-mediated SYCP2 phosphorylation affects RAD51 recruitment in TC-HR

**Figure 4.**
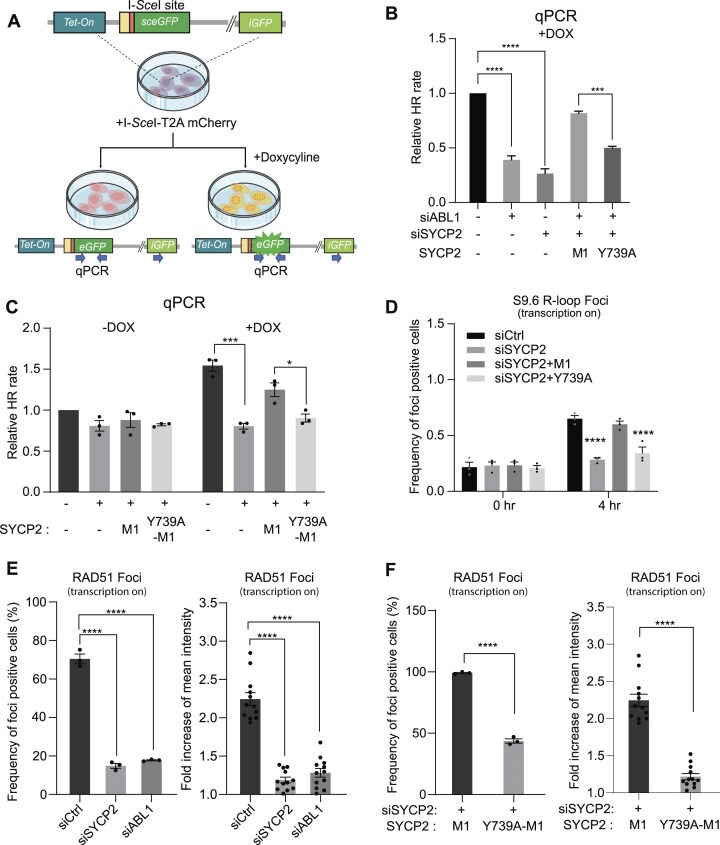
ABL1-mediated phosphorylation of SYCP2 at Y739 promotes TC-HR and RAD51 recruitment. (**A**) Schematic of the Tet-DR-GFP reporter assay used to measure HR activity under transcriptionally active (+Dox) and inactive (–Dox) conditions. (**B**) FACS-based HR quantification in U2OS Tet-DR-GFP cells with ABL1 and SYCP2 knockdown. Wild-type M1 but not Y739A mutant rescues the TC-HR defect (*n* = 3 experiments; mean ± SEM). (**C**) qPCR-based quantification of GFP repair product under + Dox and –Dox conditions. Reexpression of SYCP2-M1 restores TC-HR, while Y739A fails to fully rescue (*n* = 3 experiments; mean ± SEM). (**D**) Time-course analysis of R-loop formation following ROS-induced damage. S9.6 foci at TA-KR sites increase at 4 h in SYCP2-M1 expressing cells, but not with Y739A-M1 (*n* = 3; mean ± SEM). (**E**) Knockdown of SYCP2 or ABL1 reduces RAD51 recruitment to transcriptionally active sites. Quantified by percentage of foci-positive cells and mean fold intensity increase at TA-KR (*n* = 3–10; mean ± SEM). (**F**) SYCP2 M1 but not SYCP2-M1 restores RAD51 localization to KR damage sites in SYCP2 knockdown cells (*n* = 3–10; mean ± SEM). Statistical analysis was performed using unpaired two-tailed Student’s *t*-test. *P < .05, **P < .01, ***P < .001, ****P < .0001.

To assess the impact of SYCP2 phosphorylation on TC-HR, we utilized a Tet-DR-GFP reporter assay in U2OS cells, which allows monitoring of HR efficiency under transcriptionally active (+DOX) and inactive (−DOX) states. In this system, HR repair at the transcription site results in GFP expression, which can be quantified by flow cytometry. Additionally, both transcriptional states can be assessed using a qPCR-based assay (Fig. 4A). In siABL1-treated or siABL1- and siSYCP2-double-treated cells, we observed both significant reduction in HR efficiency during transcription. Reintroduction of the M1 fragment of SYCP2 restored HR activity, while the phosphorylation-defective mutant Y739A-M1 failed to do so, indicating the critical role of Y739 phosphorylation in HR (Fig. 4B). qPCR analysis revealed a 1.5-fold increase in HR frequency under transcriptionally active conditions compared to inactive ones (canonical HR), suggesting a preference for TC-HR. However, SYCP2 deficient cells showed no such preference (Fig. 4C). Collectively, these findings suggest that phosphorylation of SYCP2 at Y739 is essential for efficient TC-HR.

Given that SYCP2 is essential for R-loop formation, with the M1 domain specifically promoting this process [7], it is important to consider how the phosphorylation of SYCP2 influences R-loop dynamics. Phosphorylation may modulate SYCP2’s ability to facilitate R-loop formation, thereby impacting DNA repair mechanisms. Prior experiments using SYCP2 and S9.6 revealed that R-loop levels peak around 4 h after DNA damage and return to basal levels by 24 h. Consistent with our prior findings, SYCP2 knockdown markedly reduced R-loop formation at 4 h following ROS-induced DNA damage, compared to control cells. To investigate the contribution of ABL1-mediated phosphorylation, we compared M1 and its phosphorylation-deficient mutant, Y739A-M1. At 0 h post introduction, both conditions showed similarly low R-loop frequencies, but at 4 h, cells expressing M1 exhibited a significant increase in R-loop formation, while Y739A-M1 cells did not, indicating that phosphorylated SYCP2 is critical for R-loop formation in response to ROS-induced double-strand breaks (Fig. 4D). Given this, we examined whether phosphorylation influences downstream processes in TC-HR, specifically RAD51 recruitment, a critical step in TC-HR [15]. Independent knockdown of SYCP2 and ABL1 significantly reduced RAD51 localization to transcriptionally active sites (Fig. 4E). In SYCP2 pre-knockdown cells, M1 expression rescued RAD51 recruitment, whereas Y739A-M1 failed to do so, confirming that SYCP2 phosphorylation at Y739 is essential for RAD51 recruitment at DNA damage sites (Fig. 4F and Supplementary Fig. S3A). Moreover, ABL1 inhibitors, including dasatinib and imatinib, also significantly reduced RAD51 recruitment (Supplementary Fig. S3B). Together, these findings suggest that phosphorylation of SYCP2 by ABL1 is critical for its role in R-loop formation and RAD51 recruitment during TC-HR.

### ABL1 inhibitor sensitizes high SYCP2 expression platinum-resistant ovarian cancer cells *in vivo*

**Figure 5.**
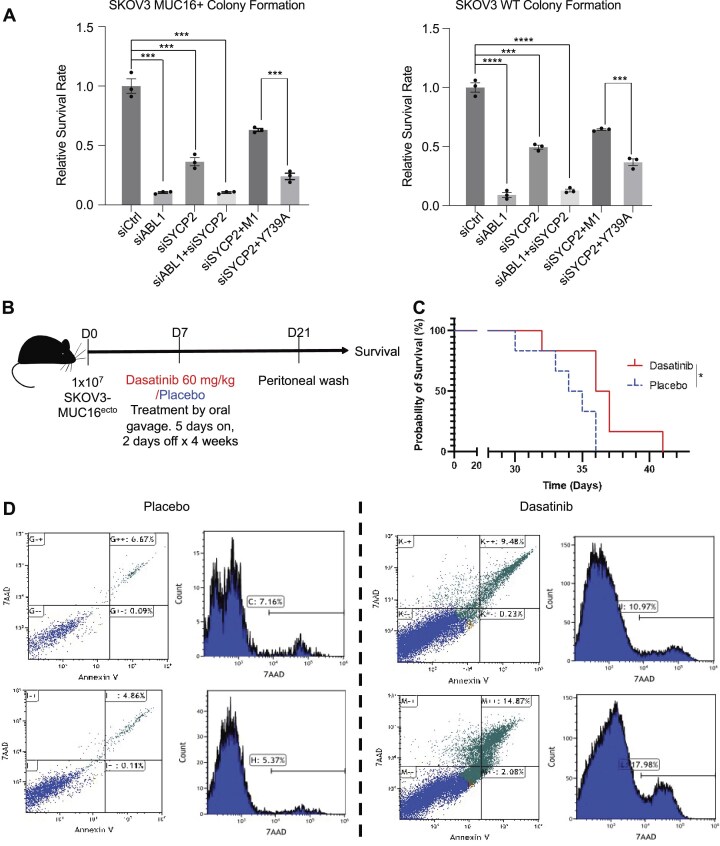
ABL1 inhibition sensitizes high SYCP2-expressing platinum-resistant ovarian cancer cells *in vivo*. (**A**) Colony formation assays in SKOV3 MUC16+ and SKOV3 WT cells transfected with the indicated siRNAs and rescue constructs. Knock down of ABL1 or SYCP2 individually reduced survival, and their combined knockdown showed no additive effect, suggesting epistasis. Reexpression of wild-type SYCP2-M1 rescued colony formation, while the Y739A mutant failed to restore viability, indicating the importance of ABL1-mediated phosphorylation at Y739 (*n* = 3 replicates; mean ± SEM). (**B**) Experimental design of the xenograft model. 1 × 10^7^ SKOV3-MUC16+ platinum-resistant ovarian cancer cells were intraperitoneally injected into NSG mice. Mice were treated with dasatinib (60 mg/kg) or placebo via oral gavage (5 days on, 2 days off) starting on day 7. (**C**) Kaplan–Meier survival curves of dasatinib versus placebo-treated mice (*n* = 6 per group). Dasatinib significantly prolonged survival (*P* = .0435, log-rank test). (**D**) Flow cytometry analysis of Annexin V and 7-AAD staining in peritoneal washes collected on day 21. Dasatinib-treated mice exhibited increased apoptotic tumor cell populations, consistent with impaired repair and enhanced cytotoxicity *in vivo*. Statistical analysis: unpaired two-tailed Student’s *t*-test unless otherwise noted. *P < .05, **P < .01, ***P < .001, ****P < .0001.

With an understanding of the role of ABL1-SYCP2 pathway in resistant ovarian cancer cells, we evaluated the translational potential of targeting SYCP2 as a biomarker and therapeutic target. The colony formation in SKOV3 wildtype or MUC16+ and OVCAR8 cells showed that lack of ABL1/SYCP2 expression or SYCP2 phosphorylation activity mediated by ABL1 would largely reduce the cancer cell survival (Fig. 5A and Supplementary Fig. S4). Notably, simultaneous knockdown of both SYCP2 and ABL1 did not further decrease colony forming rate, indicating their collaborative function within the same survival pathway. Reintroduction of the M1 domain of SYCP2 into SYCP2-knockdown cells restored colony formation to control levels, whereas the Y739A mutant failed to do so, underscoring the critical role of the M1 domain and Y739 phosphorylation site in SYCP2’s function (Fig. 5A). Extending these findings *in vivo*, we utilized a xenograft model where 10 million platinum-resistant (SKOV3-MUC16) ovarian cancer cells were intraperitoneally injected into immunocompromised NSG mice. At day 7 post-SKOV3 xenograft seeding, dasatinib (60 mg/kg) was administered by oral gavage for 2 weeks, followed by peritoneal wash at D21 in representative mice from each cohort (Fig. 5B). Survival rates of the mice in the remainder mice showed that the mice treated with dasatinib survived significantly longer than tumor-bearing mice treated with placebo (Fig. 5C). Analysis of the peritoneal washes by flow cytometry was performed to estimate *in vivo* cytotoxicity and corroborate our *in vitro* findings from Fig. 1F. We found that dasatinib treatment significantly leads to tumor cell apoptosis as shown by 7AAD and annexin V double-positive cells, corroborating the therapeutic response and prolonged survival seen in these mice (Fig. 5D). These results suggest that SYCP2 expression modulates sensitivity to ABL1 inhibition and is required for DNA repair via TC-HR, supporting a model where ABL1 inhibitors preferentially target SYCP2-high, platinum-resistant cells.

## Discussion

Our previous studies revealed the role of one such meiotic recombination protein SYCP2 in TC-HR in promoting R-loop formation and facilitating the recruitment of RAD51 to R-loops, thereby driving TC-HR at sites of transcription after damage [7]. In this study, we explored the potential role of ABL1 in TC-HR at R-loops, uncovering a novel and critical function of ABL1, which has not been extensively explored in the context of cancer biology. Previously, ABL1 was known as a non-receptor tyrosine kinase that was recognized for its roles in regulating cellular processes such as proliferation, differentiation, and DDR [10, 22–24]. Specifically in DDR, ABL1 is known for its role in regulating apoptosis, cell cycle checkpoints, and DNA repair [25], while it was unclear which function of ABL1 is critical for cell survival. Our new finding that ABL1 is actively recruited to R-loops highlights a crucial role of ABL1 in maintaining genomic stability in cancer cells. More specifically, we found that ABL1 phosphorylates SYCP2 and the phosphorylated SYCP2 is recruited at sites of R-loops and consequently enables the recruitment of RAD51, a key recombination protein, to initiate DNA repair through HR. This pathway helps to repair DSBs in transcriptionally active regions, allowing cancer cells to survive under conditions of genomic stress, such as chemotherapy and radiation. The identification of ABL1’s role in R-loop-mediated DNA repair adds a significant new dimension to our understanding of ABL1’s function in cancer biology. This study extends that understanding by showing that ABL1 is involved in a specialized form of DNA repair, linked to transcriptional activity. The phosphorylation of SYCP2 by ABL1, which stabilizes R-loops and facilitates RAD51 recruitment, suggests that ABL1 acts as a critical mediator between transcription and DNA repair in cancer cells.

Emerging evidence highlights the pivotal role of HR in cancer cells, partially through the aberrant activation of meiotic recombination proteins, which are normally restricted to germ cells [26]. In cancer cells, these proteins become co-opted to enhance the capacity of cells for DNA repair, which in turn contributes to resistance against various therapeutic strategies [7, 26, 27]. Other key meiosis-specific proteins involved in TC-HR include DMC1, a RAD51 ortholog [28]. DMC1’s aberrant expression in cancer cells contributes to proliferation and recovery from genotoxic stress by mimicking HR mechanisms, thus aiding in maintaining genomic stability under therapeutic stress [29]. Conversely, HORMAD1, which are ectopically expressed in cancers such as lung adenocarcinoma and squamous cell carcinoma, promotes genomic instability [30]. HORMAD1 has been identified as a driver of genome instability in triple-negative breast cancers by suppressing RAD51-dependent HR, leading to increased reliance on nonhomologous end joining pathways [31]. Another synaptonemal complex member, SYCP3, was shown to inhibit RAD51 recruitment to DNA damage sites by interacting with BRCA2, resulting in defective sister-chromatid recombination, chromosomal instability, and heightened sensitivity to DNA-damaging agents [32]. The exploitation of these proteins by cancer cells to survive in hostile therapeutic environments presents these proteins as prime targets for intervention, particularly in cancers where DNA damage is a core aspect of treatment resistance. It is possible that SYCP2 might cooperate or be mutually exclusive with these meiotic proteins, forming a regulatory network that enables cancer cells to exploit meiotic recombination mechanisms for survival. Future studies for understanding this relationship are crucial for identifying personalized therapeutic strategies to disrupt cancer cell survival mechanisms.

This uncovered function of ABL1 at sites of R-loops has significant implications for cancer therapy, particularly in cancers where transcriptional dysregulation and DNA repair are intertwined. For example, ovarian cancer cells with high SYCP2 expression and R-loop accumulation are more resistant to platinum-based therapies due to their enhanced DNA repair capabilities. Although ABL1 is ubiquitously expressed across cell types, studies including TCGA data indicate that in certain ovarian cancer subtypes—particularly epithelial ovarian carcinomas with poor prognosis—ABL1 expression and kinase activity may be elevated [12, 33–35]. While not frequently mutated or genomically amplified, ABL1’s nuclear localization and activity are often enhanced in response to genotoxic stress. This stress-induced activation is consistent with its functional role in transcription-coupled DNA repair pathways, including the SYCP2-dependent mechanism described in this study. By targeting ABL1’s role in R-loop-dependent DNA repair, particularly through the use of TKIs such as imatinib or dasatinib, we can disrupt this pathway and render cancer cells more vulnerable to chemotherapy. This disruption of ABL1-mediated SYCP2 phosphorylation impairs the recruitment of RAD51 to R-loops, leading to defective HR and increased genomic instability, which ultimately sensitizes cancer cells to treatment. While our in vivo experiments suggest that dasatinib can effectively target SYCP2-high tumors, it is important to acknowledge the limitations of this model in definitively establishing SYCP2 dependence for dasatinib response. In particular, our xenograft studies utilized SYCP2-high SKOV3-MUC16 + cells, which reliably form tumors in mice, whereas SYCP2-low cells demonstrate poor tumorigenicity and are not suitable for comparative xenograft analysis. This technical limitation precludes direct *in vivo* comparison of dasatinib response between SYCP2-high and SYCP2-low tumors. Nevertheless, our in vitro experiments robustly demonstrate that SYCP2 expression modulates sensitivity to ABL1 inhibition and is required for DNA repair via TC-HR, supporting a model in which ABL1 inhibitors preferentially target SYCP2-high, platinum-resistant cancer cells.

However, this discovery also raises several questions about the broader implications of ABL1’s activity at R-loops in other cancers. For example, how broad ABL1’s involvement in R-loop-associated DNA repair varies across different cancer types, and if there are other substrates or cofactors that modulate ABL1’s activity in this context. Moreover, it is still unclear how ABL1 is recruited to R-loops. Understanding the upstream and downstream signals that regulate ABL1’s localization and activity at R-loops could reveal additional therapeutic targets and help refine strategies for targeting the ABL1-SYCP2 axis in cancer. ABL1 is a kinase with diverse roles in cellular processes and its functions are still not fully understood, inhibiting ABL1 could impact not only SYCP2 but also other substrates within the DDR network. In addition to SYCP2, ABL1 also phosphorylates other DNA repair proteins, including RAD52 and RAD51. Yuan *et al.* first reported this modification at tyrosine 104 (Y104) of RAD52, demonstrating that this phosphorylation is important for RAD52 foci formation and DNA repair function [35]. While our findings suggest that SYCP2-mediated TC-HR operates independently of RAD52 in ovarian cancer, the potential cooperation between these ABL1 substrates—especially under stress conditions—merits further investigation. Moreover, continued analysis on the broader role of SYCP2 in DNA repair could provide deeper insights into how meiotic proteins contribute to therapeutic resistance and identify additional therapeutic targets. Regardless of these questions, our discovery of the function of ABL1-SYCP2 at R-loops opens new avenues for exploring the intersection of transcription and DNA repair in cancer. Understanding these pathways in greater detail will be crucial for developing novel therapeutic strategies that exploit the vulnerabilities of transcription-coupled DNA repair mechanisms in cancer.

Our study uncovers the underlying mechanism by which SYCP2 influences the DDR pathway through ABL1-mediated phosphorylation. Previous studies have shown the role of ABL1 for the regulation of DNA damage repair [24]; however, this study reveals ABL1’s role in R-loop-dependent DNA damage repair as ABL1 is recruited to sites of R-loops. This discovery of SYCP2’s mechanism in DDR provides novel insight that TKIs can impair transcription-associated homologous recombination repair through ABL1-mediated SYCP2 phosphorylation, thus a potential inhibitory effect on platinum-resistant ovarian cancer cells. Targeting ABL1 has been effective for leukemia [36], but its application for other cancers is limited [37]. As such, our findings expand the application of targeting ABL1 for other cancer types and could be an option when formulating drug combinations for platinum-resistant ovarian cancer.

## Supplementary Material

zcaf031_Supplemental_File

## Data Availability

All data are available in the main text or the supplementary material.
