# Supplementary material for: ABL1-mediated tyrosine phosphorylation of SYCP2 contributes to transcription-coupled homologous recombination and platinum resistance in ovarian cancer
Source: NAR Cancer. 2025 Sep 3;7(3):zcaf031. doi: 10.1093/narcan/zcaf031 (PMC12409405; doi:10.1093/narcan/zcaf031)
Supplement: zcaf031_Supplemental_File [file zcaf031_supplemental_file.docx]

**ABL1-mediated tyrosine phosphorylation of SYCP2 contributes to transcription-coupled homologous recombination and platinum resistance in ovarian cancer**

Boya Gao^1,2^*, Xudong Wang^2^*, Melissa Long^1,2^, Fengqi Zhang^1,2^, Yumin Wang^2^, Raj Kumar^3,4^, Irva Veillard^3,4^, Bo R. Rueda^5,6,7^, Oladapo Yeku^3,4^, Li Lan^1,2^†

^1^Department of Molecular Genetics and Microbiology, Duke University School of Medicine, 213 Research Drive, Durham, NC, 27710, USA.

^2^Massachusetts General Hospital Cancer Center, Harvard Medical School, 13th Street, Charlestown, MA, 02129, USA.

^3^Division of Hematology-Oncology, Massachusetts General Hospital, 55 Fruit Street, Boston, MA, 02114, USA.

^4^Department of Medicine, Massachusetts General Hospital, 55 Fruit Street, Boston, MA, 02114, USA.

^5^Division of Gynecologic Oncology, Department of Obstetrics and Gynecology, Massachusetts General Hospital, 55 Fruit Street, Boston, MA, 02114, USA.

^6^Obstetrics, Gynecology and Reproductive Biology, Harvard Medical School, 25 Shattuck Street, Boston, MA, 02115, USA.

^7^Vincent Center for Reproductive Biology, Department of Obstetrics and Gynecology, Massachusetts General Hospital, 55 Fruit Street, Boston, MA, 02114, USA.

* authors contributed equally to this work

† To whom correspondence should be addressed: Dr. Li Lan Department of Molecular Genetics and Microbiology, Duke University School of Medicine; 213 Research Drive, Durham, NC, 27710, USA; Phone: 919-613-8626; E-mail: li.lan@duke.edu

**One sentence summary: ABL1 phosphorylates SYCP2 in DNA repair and promotes platinum resistance.**


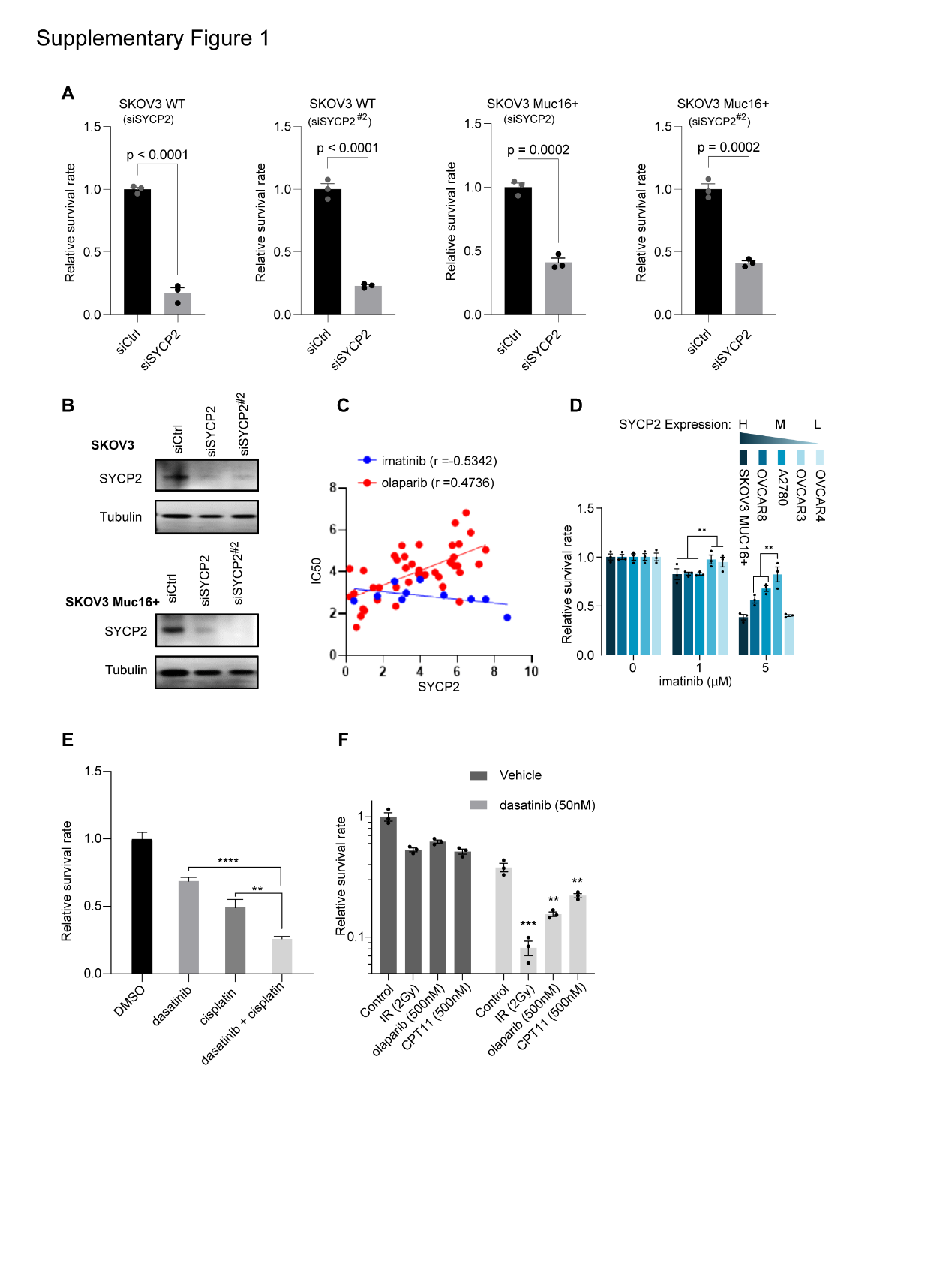


**Supplementary Figure 1. SYCP2 expression modulates sensitivity to ABL1 inhibitors in ovarian cancer cells.** **A.** Colony formation assays in SKOV3 WT and SKOV3 MUC16⁺ cells upon SYCP2 knockdown using two independent siRNAs. (n = 3 repeats, mean ± SEM). **B.** WB of SYCP2 in SKOV3 WT and SKOV3 Muc16+ cells with siSYCP2 or siSYCP2^#2^ treatment. **C.** Correlation of SYCP2 expression with drug IC50 in ovarian cancer cell lines based on GDSC database. imatinib (r = –0.5342); olaparib (r = 0.4736). **D.** Relative survival of a panel of ovarian cancer cell lines treated with increasing doses of imatinib (0–5 µM). (n = 3 repeats, mean ± SEM). **E.** Combination treatment of dasatinib and cisplatin in SKOV3 cell compared to single agents (n = 3, mean ± SEM). **F.** Combination treatment of dasatinib and multiple genotoxic agents (IR, olaparib, CPT11) in SKOV3 cells compared to single agents (n = 3, mean ± SEM). Statistical analysis was performed with unpaired two-tailed Student’s t-test. *p < 0.05; **p < 0.01; ***p < 0.001; ****p < 0.0001.


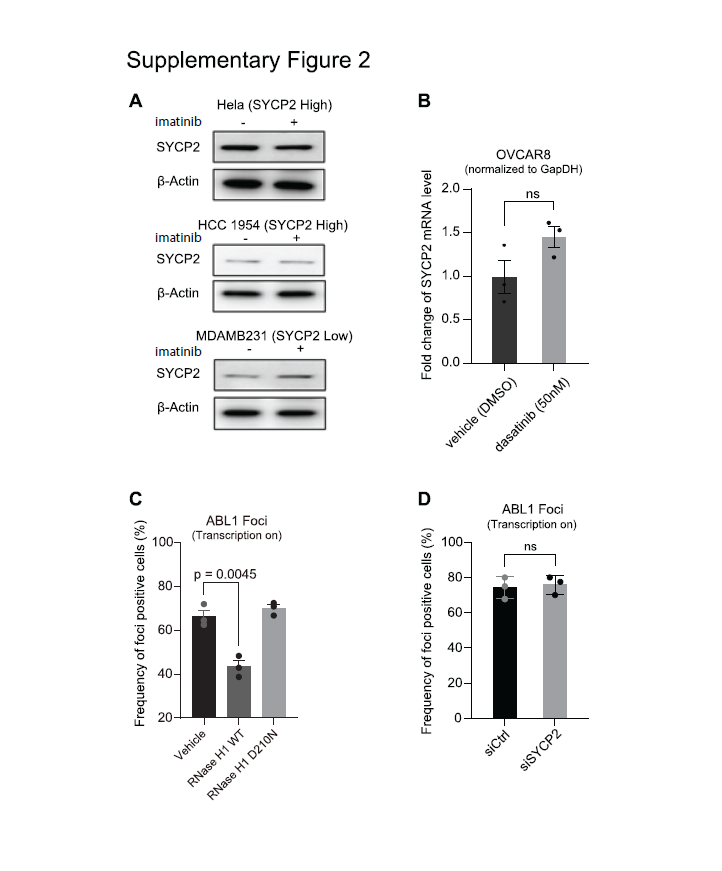


**Supplementary Figure 2. ABL1 inhibition does not alter SYCP2 expression or ABL1 localization to R-loops.** **A.** Western blot of SYCP2 protein levels in HeLa, HCC1954 (SYCP2-high), and MDA-MB-231 (SYCP2-low) cells treated with or without 1 µM imatinib. **B.** RT-qPCR analysis of SYCP2 mRNA levels in OVCAR8 cells treated with 50 nM dasatinib for 48 hours. (n = 3 replicates, mean ± SEM). **C.** Frequency of ABL1 foci positive cells with expression of RNase H1 WT or D210N catalytic defective mutant at TA-KR. (n = 3 groups, mean ± SEM). **D.** Frequency of ABL1 foci positive cells treated with siCtrl or siSYCP2 at TA-KR. (n = 3 groups, mean ± SEM). Statistical analysis was performed with unpaired two-tailed Student’s t-test. ns: not significant.


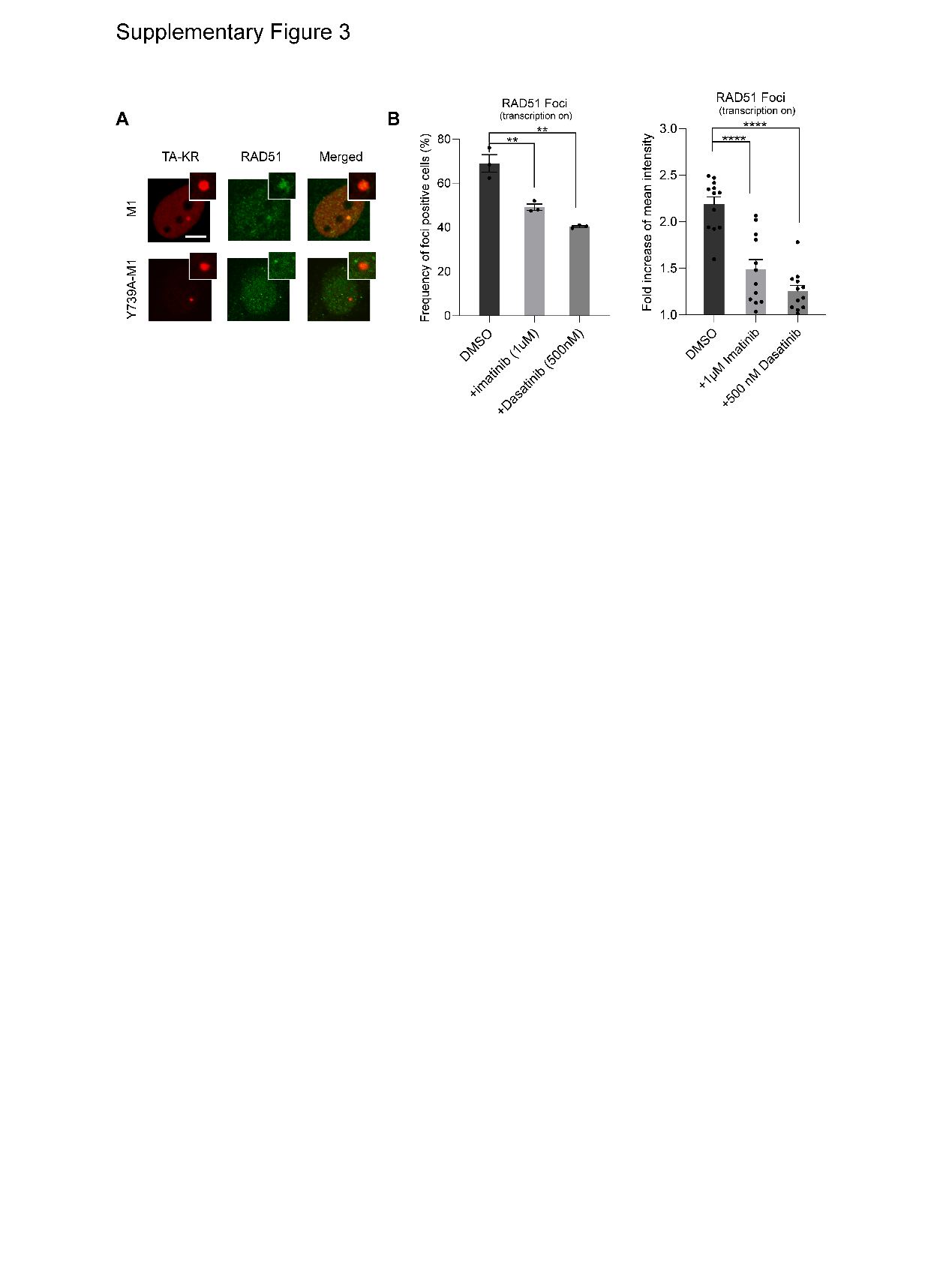
**Supplementary Figure 3. ABL1 inhibition disrupts RAD51 recruitment at R-loops via SYCP2 Y739 phosphorylation.** **A.** Representative images of RAD51 foci in U2OS-TRE cells transfected with TA-KR and either GFP-M1 or GFP-Y739A-M1, 30 min post damage. **B.** Left: Frequency of RAD51 foci positive cells at TA-KR under DMSO, imatinib (1 μM), or dasatinib (500 nM) treatment. Right: Fold increase of mean intensity of RAD51 foci at TA-KR sites compared to nuclear background (n = 10 cells, mean ± SEM). Statistical analysis was performed using an unpaired two-tailed Student’s t-test. **p < 0.05; **p < 0.01; ****p < 0.0001.


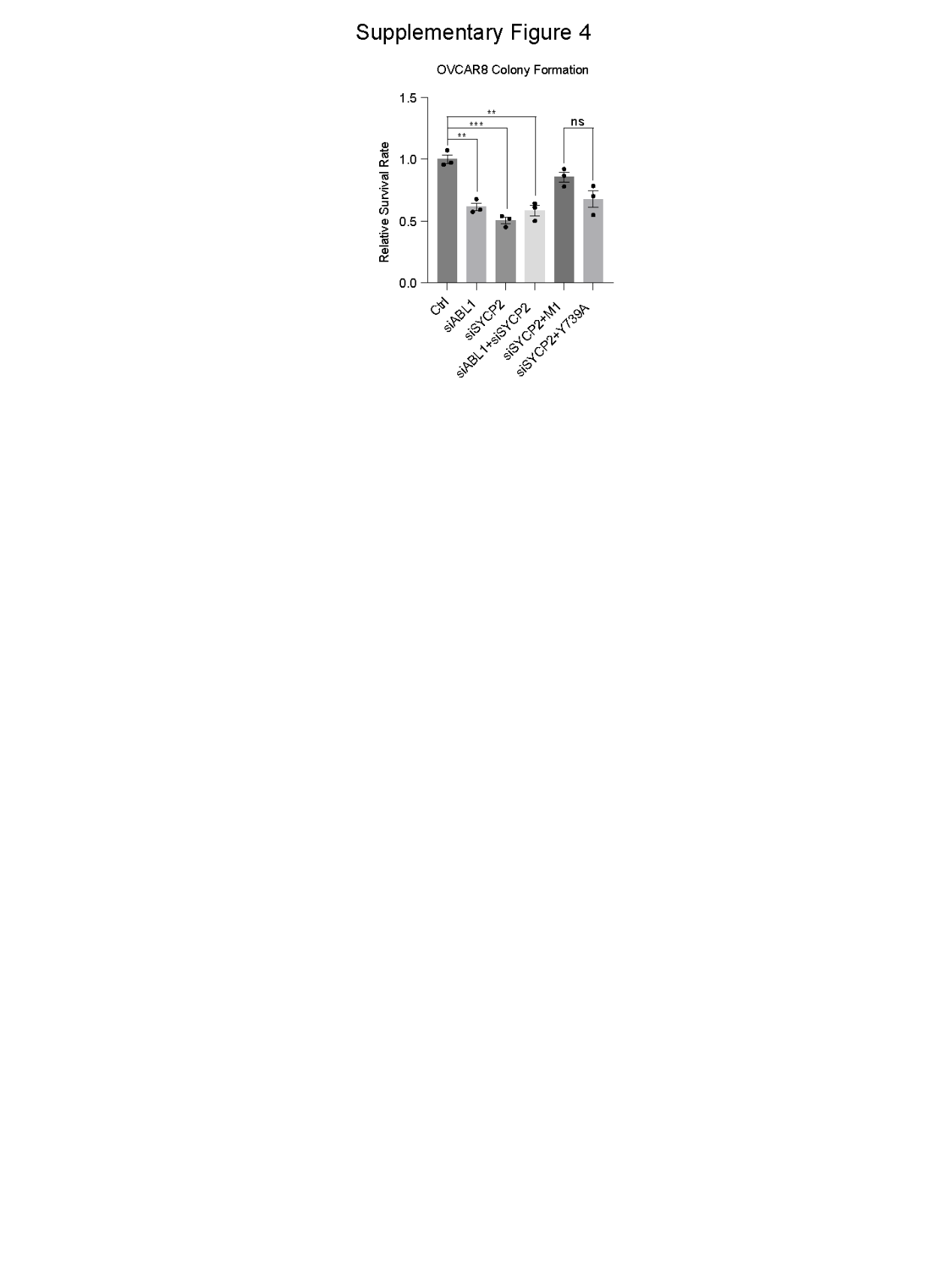
**Supplementary Figure 4. Phosphorylation-defective SYCP2 Y739A mutant fails to restore cell survival in SYCP2-depleted ovarian cancer cells.** OVCAR8 cells were treated with siCtrl, siABL1, siSYCP2, or a combination of siABL1 and siSYCP2. Cells were also rescued with either GFP-SYCP2-M1 or the phosphorylation-defective mutant GFP-SYCP2-Y739A following siSYCP2 knockdown. Colony formation assays were performed, and relative survival rate was quantified (n = 3, Mean ± SEM). Statistical analysis was performed using an unpaired two-tailed Student’s t-test. **p < 0.01; ***p < 0.001; ns, not significant.
